# Effects of Rhythm Control versus Rate Control on Atrial Fibrillation and Frailty Development in Older Patients: Protocol for the Cardioversion of Atrial Fibrillation and Frailty in the Elderly (CAFFE) Study, an Observational, Prospective Cohort Study

**DOI:** 10.2196/92572

**Published:** 2026-09-29

**Authors:** Elisa Berni, Veronica De Angelis, Emanuele Santamaria, Alessandro Mengozzi, Claudia Di Serio, Giulia Spanalatte, Michele Biagioli, Arianna Tariello, Marta Niccolini, Camilla Cagnoni, Djullye Miduri Da Silva Nakano, Giada Alla Viligiardi, Marco Capacci, Andrea Ungar, Giancarlo La Marca, Igor Diemberger, Agostino Virdis, Stefano Fumagalli

**Affiliations:** 1 Department of Experimental and Clinical Medicine, University of Florence, Viale Pieraccini, 6, Florence, 50139, Italy, 39 055-2751821; 2 Department of Medical and Surgical Sciences, University of Bologna, Bologna, Italy; 3 Department of Clinical and Experimental Medicine, University of Pisa, Pisa, Italy; 4 Department of Neuroscience and Medical Genetics, Newborn Screening, Clinical Biochemistry and Clinical Pharmacy Laboratory, Meyer Children's Hospital IRCCS, Florence, Department of Experimental and Clinical Biosciences, University of Florence, Florence, Italy

**Keywords:** atrial fibrillation, disability, frailty, Comprehensive Geriatric Assessment, older patients, rhythm-control therapy

## Abstract

**Background:**

Atrial fibrillation (AF), highly prevalent in older individuals, is associated with frailty and disability. AF therapy is based on rhythm control (RhythmC) to restore sinus rhythm or rate control (RateC) to lower the heart rate. Published guidelines do not indicate a preference for one over the other for older people.

**Objective:**

This study aims to evaluate whether RhythmC is more effective than RateC in hindering the progression of frailty and disability.

**Methods:**

The Cardioversion of Atrial Fibrillation and Frailty in the Elderly (CAFFE) study is a prospective, multicenter, observational cohort study that includes 4 groups of patients: (1) adults age 75 years or older undergoing RhythmC treatment; (2) adults aged 65 to 74 years undergoing RhythmC treatment; (3) adults 65 years or older undergoing RateC treatment; and (4) adults 65 years or older without overt chronic conditions as the control group (n=40 in each group). At baseline, all participants will undergo a Comprehensive Geriatric Assessment (CGA) and analyses of inflammation and metabolomics. At home, a device will evaluate each participant’s heart rhythm, activity profile, and sleep. After 1 month, the CGA will be repeated.

**Results:**

The CAFFE study was funded as a Research Project of National Interest by the Italian Ministry of University and Research (October 2023). Ethical committee final approval was given in June 2024. In October 2025, the coordinating center had enrolled 31 of 40 patients with AF (mean age 78, SD 9 years; 8 [25.8%] women; mean CHA_2_DS_2_-VA score 4.2, SD 1.2; 13 [43.3%] robust participants). All centers are now completing the enrollment (103/160 participants), and the analysis of inflammatory mediators has started. The results are expected to be published in summer 2027.

**Conclusions:**

The CAFFE study will evaluate the effectiveness of AF management strategies in older patients. If RhythmC can slow the progression of frailty and disability, specific clinical trials should more definitively address this knowledge gap.

## Introduction

### Atrial Fibrillation and Frailty

Atrial fibrillation (AF) is the most frequent sustained arrhythmia affecting older people, with a prevalence as high as 35% in subjects older than 85 years [1]. The aging process, the improvement of acute cardiovascular care, and the higher exposure to risk factors are responsible for the progressive increase in older individuals with arrhythmia [1]. Patients with AF exhibit a disorganization in the electrical activity of the cardiac atria, leading to irregular contractions of the ventricles and resulting in a reduction in cardiac output and, thus, in an altered blood supply to all organs. This condition can lead to symptoms such as palpitations, fatigue, and dyspnea, and it is usually correlated with an increased risk of stroke, heart failure, and dementia, particularly in the aging population, where AF can provide the basis for the development of frailty and disability [2-4] (Figure 1).

**Figure 1.**
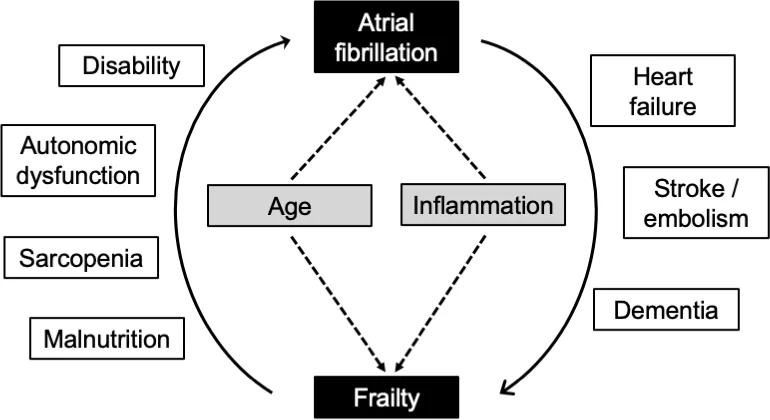
The bidirectional association between atrial fibrillation (AF) and frailty.

The primary contributors to and risk factors for the onset and recurrence of the disease can be ascribed to foci located in the pulmonary veins and to cardiovascular and systemic diseases, including diabetes, obesity, obstructive sleep apnea, and chronic kidney and respiratory diseases. These conditions are strongly represented in older patients, who are characterized by multimorbidity and a state of low-grade inflammation, further enhancing the risk of AF onset [5].

As the global population ages, disability associated with AF increases. This is related to a decline in functional performance, a reduction in quality of life, and the development of frailty itself, leading to a vicious cycle where frail individuals are more prone to developing AF, which in turn further exacerbates frailty and associated symptoms, ultimately impacting overall quality of life [6,7]. From a molecular point of view, fibrosis development and changes in the autonomic nervous system activity favor AF onset, while sarcopenia is related to the worsening of frailty [7].

Frailty is a multifactorial syndrome caused by a reduction in the physiological reserve and capacity to resist stressful events. It appears to be age related, with a prevalence as high as 20% in patients older than 80 years. It can be viewed as a byproduct of the natural and irreversible decline associated with aging in various physiological systems [8].

Frailty itself, as an age-related syndrome, may be attributed to chronic low-grade systemic inflammation [5]. Chronic inflammation induces several changes in biological processes at both the cellular and systemic levels, such as DNA damage, mitochondrial dysfunction, and defective autophagy, leading to metabolic impairment and a derangement in energy production and use [9,10].

The heart, which constantly requires energy, is particularly affected by the aging process and is prone to sustaining damage due to its high mitochondrial activity; this correlation could explain the high incidence of cardiovascular diseases in older individuals [9,10].

### AF Treatment

To date, 2 main approaches exist for the treatment of AF, one based on rate control (RateC) and the other based on rhythm control (RhythmC). The RateC approach aims to regulate the heart rate without interrupting arrhythmia, while the RhythmC strategy is intended to restore sinus rhythm with electrical cardioversion (ECV) or pharmacological cardioversion (PCV) and to prevent recurrences with antiarrhythmic drugs or AF ablation [11,12].

The literature includes some clinical trials, such as the AFFIRM study, that suggest that the use of RhythmC treatment for patients with AF who are elderly and frail is associated with higher mortality rates [13]. However, some recent evidence suggests that there are advantages to pursuing RhythmC in older individuals. Indeed, observational studies have found that the restoration and maintenance of sinus rhythm were correlated to a reduction in mortality and ischemic stroke [14] and that the cognitive performance of older patients was better when treated with a RhythmC strategy than with a RateC strategy [15].

Additionally, AF leads to a decrease in cardiac output due to the shortening of diastole and the loss of atrial systole, which, in turn, can result in relevant cognitive changes through a reduction in cerebral blood flow [3,16]. Sinus rhythm restoration has been shown to improve brain perfusion [17]. Finally, the recent EAST-AFNET trial compared the RhythmC strategy to the usual RateC therapy and found that the implementation of the RhythmC strategy within 12 months of arrhythmia onset correlated with a lower risk of adverse cardiovascular outcomes (ie, the composite of cardiovascular mortality, stroke, and hospitalizations for heart failure and acute coronary syndromes) [18]. These observations suggest that the interruption of arrhythmia can hinder functional decline and the development of frailty in an older population with AF.

### Objectives

The preceding literature review demonstrates that the findings related to this topic are controversial. Accordingly, which therapeutic approach (ie, the RateC or the RhythmC strategy) should be chosen for older patients with AF, especially those who are complex and frail, is still not clear [2,11,12], and this is the reason why this study was planned. In particular, this project aims to evaluate whether RhythmC is more effective than RateC in controlling arrhythmia and hindering the progression of frailty and disability.

## Methods

### Design of the Cardioversion of Atrial Fibrillation and Frailty in the Elderly (CAFFE) Study

The CAFFE study is a prospective, multicenter, observational cohort study aimed at clarifying some of the knowledge gaps regarding the management of AF in older patients and providing evidence to support therapeutic decisions to improve clinical outcomes and quality of life. The effects of the reduction of arrhythmia burden on disability and frailty in the elderly are largely unexplored [2]. Specifically, the goal of this study is to clarify whether, compared to the RateC approach, the RhythmC strategy can exert a more beneficial effect on frailty, functional performance, quality of life, and the neurocognitive profile of older individuals with persistent AF (Figure 2).

**Figure 2.**
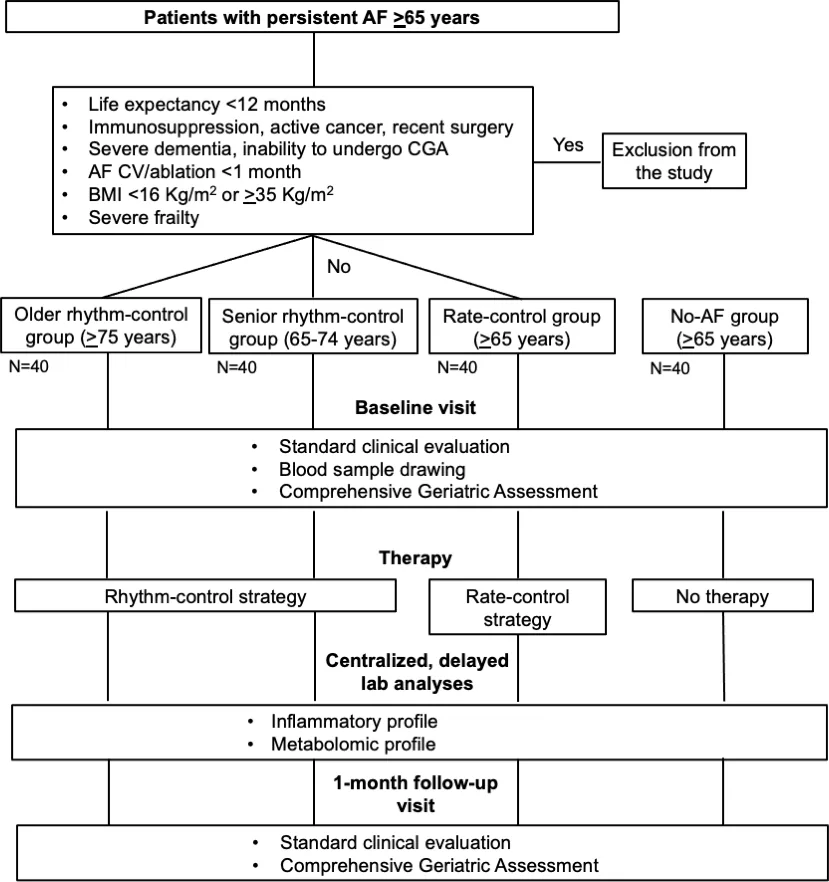
The Cardioversion of Atrial Fibrillation and Frailty in the Elderly (CAFFE) study flowchart. AF: atrial fibrillation; CGA: Comprehensive Geriatric Assessment; CV: cardioversion; lab: laboratory.

### Recruitment

The CAFFE study will enroll a cohort of 120 patients with AF and 40 healthy control participants during a 13-month window after protocol approval by the ethics committees of the participating centers. The 120 patients with AF will be divided into 3 groups based on age and treatment criteria. Participants will be enrolled in an outpatient setting and will be evaluated at baseline and at the 1-month follow-up. Participants screened during hospitalization will be included in this study at least 1 month after discharge home and after the exclusion of a persistently active acute condition.

To be included in this study, patients must have had a diagnosis of AF determined by an electrocardiogram (EKG), a Holter EKG, or an implantable device in the preceding 12 months. The exclusion criteria are (1) having any clinical condition associated with a life expectancy of less than 12 months, (2) being in ongoing therapy with immunosuppressant drugs, (3) having active cancer and undergoing radiotherapy or chemotherapy, (4) having recently (<1 month previously) had major surgery, (5) having severe cognitive decline or an inability to follow the therapeutic schemes, (6) being physically unable to undergo the Comprehensive Geriatric Assessment (CGA), (7) having permanent AF, (8) having received any attempt of ablation/cardioversion of AF in the previous month, (9) having cachexia/extreme thinness (BMI<16 kg/m^2^) or obesity (BMI>35 kg/m^2^), (10) having a severe preexisting frailty, and (11) refusing to participate.

Three centers, strictly adhering to current guideline recommendations for the overall management of patients [12], will take part in this study following their usual clinical practice. In particular, the Geriatric Cardiology Unit of the University of Florence will preferentially enroll patients in the older RhythmC group, the cardiology unit of the University of Bologna will enroll patients in the senior RhythmC group, and the geriatric unit of the University of Pisa will enroll patients in the RateC group. The control group population will be enrolled by all centers during routine outpatient activity.

### Study Procedures

#### Baseline

At baseline, each participant will undergo a standard clinical evaluation in a fasting state, including an EKG and echocardiogram. This will be followed by the administration of the CGA and drawing of blood samples. Immediately after, patients with AF, who must have been anticoagulated for at least 4 weeks, will be treated according to current guidelines following the RhythmC or the RateC strategy. Patients undergoing RhythmC treatment may be treated with ECV, PCV, or AF ablation. Patients following the RateC strategy will be prescribed beta-blocking agents, calcium antagonists, and digoxin.

#### Follow-up

At the 1-month follow-up, each participant will have a standard clinical visit and undergo a repeat CGA evaluation. Such a short follow-up period is needed to optimize the procedures among the 3 different centers and to account for the pathophysiological motivations that follow. Previous observational studies found that an effective RhythmC strategy is associated with a 56% increase in cardiac output at the 4-week evaluation. Such an improvement was caused by the progressive return of left atrial mechanical activity due to atrial myopathy reversal [19]. Furthermore, sinus rhythm restoration was associated with a rapid (6 hours) improvement in left ventricular performance as measured by longitudinal strain analysis [20]. Other studies found that after ECV, brain perfusion was significantly improved within a time window ranging between 4 and 10 weeks [21]. Finally, as illustrated in detail below, frailty measures, which include specific items about exhaustion, sedentary behavior, and physical activity, are greatly influenced by cardiac performance [22,23]. According to the observational design of this protocol, blood samples will not be drawn at the end of the follow-up (Figure 2).

### The CGA

#### Overview

The CGA will evaluate different features of patients using selected tests that are well established, extensively validated, and widely used in current clinical practice.

#### Cognitive function

The cognitive function of patients will be studied using the Mini-Mental State Examination (MMSE), a neuropsychological test employed to screen cognitive deficits, specifically those associated with dementia. The test consists of several sections that evaluate orientation in time and space, short-term memory and recall, attention, calculation, language comprehension, and executive functions. The maximum score of the test is 30 points, with values less than 24 suggesting potential cognitive impairment [24].

#### Disability

The presence of disability will be assessed by 2 widely used functional evaluation scales: the Katz Basic Activities of Daily Living (ADL) scale and the Lawton Instrumental Activities of Daily Living (IADL) scale [25,26]. The ADL scale measures individual ability to perform basic self-care activities, such as bathing, dressing, toileting, mobility, continence, and feeding. It provides a simple assessment of functional independence, with scores ranging from 0 (completely dependent) to 6 (fully independent) [25]. The IADL scale evaluates more complex daily activities that require cognitive and physical skills, including using the telephone, managing finances and medications, shopping, meal preparation, housekeeping, laundry, and transportation. This scale helps to identify early functional decline, with scores ranging from 0 (complete dependence) to 8 (full independence). Together, the 2 scales provide a comprehensive assessment of a patient’s functional status, allowing physicians to identify different levels of disability and support needs in everyday life.

#### Depressive Symptoms

The 15-item Geriatric Depression Scale (GDS-15) will be used to assess depressive symptoms [27,28]. It consists of 15 yes/no questions designed to evaluate mood, motivation, and overall psychological well-being. The total score ranges from 0 to 15, with higher scores indicating a larger presence of depressive symptoms. A score equal to or greater than 5 is associated with a depressive mood. Due to its simplicity, it is particularly suitable for older patients, including those with mild cognitive impairment [27].

#### Health-Related Quality of Life

Health-related quality of life will be explored with the 12-item Short Form Health Survey (SF-12), which consists of 12 questions covering 2 main areas: physical health (such as functioning and pain) and mental health (such as emotional well-being and social functioning) [29]. Accordingly, 2 summary scores, ranging between 0 and 100 (where 100 is the best performance), will be provided for both the physical and the mental components of the instrument. The SF-12 is the shortened version of the 36 -item Short Form Health Survey and is commonly used to assess the impact of diseases on health-related quality of life [29].

#### Frailty

The presence of frailty at baseline will be evaluated with the Frail Non-Disabled (FiND) questionnaire, which consists of 5 questions, 2 specifically aimed at identifying individuals with disability (“Have you any difficulties at walking 400 meters?” and “Have you any difficulties at climbing up a flight of stairs?”) and 3 that evaluate specific components of the frailty syndrome: weight loss (“During the last year, have you involuntarily lost more than 4.5 kg?”), exhaustion (“How often in the last week did you feel that everything you did was an effort or that you could not get going?”), and sedentary behavior (“Which is your level of physical activity?”). A patient is considered disabled if 1 of the conditions exploring disability is present; frailty is defined by an abnormal answer to 1 of the related items and disability is absent. A participant is evaluated as robust when no altered conditions are found [22]. To corroborate the presence of frailty, we will also observe whether a frailty phenotype exists based on 5 different conditions (ie, weight loss, exhaustion, physical activity, walk time, and grip strength). A prefrailty or a frailty phenotype is present if, respectively, 1 to 2 or 3 or more altered characteristics were found [23]. Finally, we will also use the Clinical Frailty Scale, a widely used summary instrument for clinicians, which was originally developed for the Canadian Study of Health and Aging. The scale ranges from level 1 (very fit) to level 9, with 8 indicating very severe frailty and 9 indicating terminal illness. For each level, a simple image and short description are provided to guide patient classification [30].

#### Physical Performance

Physical performance will be assessed using the Short Physical Performance Battery (SPPB), an instrument exploring 3 different functional components, namely, balance (ie, the ability to stand with the feet in side-by-side, semitandem, and tandem positions), walking speed (ie, the time necessary to walk 4 meters), and strength and endurance (ie, the time necessary to rise from a chair and return to the original position 5 times without using the arms). According to the time required to complete each task, a score ranging from 0 to 4 (where 4 is the best performance) is assigned to the patient. Consequently, the SPPB total score ranges from 0 to 12, with a score less than or equal to 6 identifying participants at higher risk of disability and mortality [31].

### Evaluation of Daily Activity, Sleep, and AF Characteristics or Relapse

After baseline evaluation, at discharge from the outpatient clinic, each participant will receive a multipurpose recorder (RootiRx, Rooti Labs Ltd) equipped with 3 accelerometers positioned on the body to acquire 48 hours of signal (ie, EKG, day and night physical activity, sleep components, and rhythm and sleep abnormalities). The device has an EKG sampling rate of 500 Hz (24-bit resolution). All stored data will be sent through a web-based application to a cloud platform and analyzed using AI via the RootiRx system [32]. For the purposes of this project, we decided to evaluate the information from the second day of recording to avoid the effects of anesthetic drugs. To ensure homogeneous assessment, we will follow this directive for all enrolled individuals. For each participant, we will evaluate the persistence and characteristics of sinus rhythm or the effectiveness of RateC in controlling AF; the period spent standing, the period spent sleeping, including the proportions of light (stages N1 and N2), deep (stage N3), and rapid eye movement sleep, and sleep efficiency (ie, the ratio between sleep time and total time spent in bed); and the Chest Effort Index (the mean number of abnormal chest efforts per hour of sleep), a measure of obstructive sleep apnea.

### Inflammatory Mediators and Metabolic Evaluation

Each participant’s inflammatory status and metabolic profile will be explored through the analysis of venous blood samples, which will be drawn at baseline in a fasting state, before clinical evaluation. Plasma will be immediately separated and frozen at −80 °C after centrifugation for 15 minutes (2500 rpm, 4 °C) until final analysis, which will be performed centrally at the Laboratory of the University of Florence.

The inflammatory profile will be assessed by measuring plasma levels of interleukin-6 (IL-6), tumor necrosis factor-α (TNF-α), and osteoprotegerin (OPG) using commercially available enzyme-linked immunosorbent assay kits.

The study of cytokine concentrations provides important information on the progression of AF, the risk of related cardiovascular and systemic complications, and the degree of frailty [10]. AF is closely associated with chronic inflammation [5,33]; proinflammatory cytokines such as IL-6 and TNF-α play a key role in endothelial dysfunction, atrial fibrosis, and disease progression [34,35]. Elevated levels of these markers are linked to an increased risk of AF persistence and adverse cardiovascular outcomes [33]. As previously mentioned, chronic inflammation is also a hallmark of frailty, contributing, through sarcopenia development, to functional decline and an increased vulnerability to stressors [36].

OPG is a glycoprotein, also known as tumor necrosis factor receptor superfamily member 11B; it can inhibit the receptor activator nuclear factor kappa-B ligand and the TNF-related apoptosis-inducing ligand, pathways involved in apoptosis. OPG is involved in bone metabolism, and it has been identified as a potential biomarker of frailty. Elevated OPG levels, indicative of a higher inflammatory response, are associated with an increased risk of cardiovascular disease, AF, cancer, immune system disorders, osteoporosis, and fractures [37].

Metabolomics provides essential information to better understand the biology of the disease and to identify diagnostic and therapeutic biomarkers. For metabolomic analyses, a targeted Biocrates AbsoluteIDQ p180 assay (Avance BioSciences), combined with liquid chromatography and tandem mass spectrometry, will be used. This method will allow the quantification of 188 metabolites, including amino acids, biogenic amines, monosaccharides, lipids, and acylcarnitine. Metabolomics should provide crucial insights into the alterations associated with oxidative stress, energy metabolism, frailty, and AF in older adults.

### End Points of the Study

The primary end point of this study is to evaluate at the 1-month follow-up visit whether an improvement in functional status in patients treated with the AF RhythmC strategy is greater than that observed in patients following the RateC strategy. Using the SPPB, our measure of physical performance, we will define an increase in each single score point as indicative of a positive change.

The secondary end points of this study are as follows. First, we will evaluate whether the reduction in frailty in participants undergoing RhythmC therapy is greater than that observed in patients undergoing RateC therapy. When specifically evaluating frailty using the FiND questionnaire, an improvement will be considered the transition from the disabled or the frail category to a better category (ie, from disabled to not disabled status and from frail to robust status). For the frailty phenotype, a patient will be considered improved when reaching at least prefrailty from the frailty phenotype or robustness from a prefrail condition. Finally, using the Clinical Frailty Scale, improvement will be defined as achievement of a better-performing status, indicated by a lower level of the scale (eg, from level 4, “Vulnerable,” to level 3, “Managing Well”). Second, we will evaluate changes between baseline and follow-up of depressive symptoms, the neurocognitive profile, and health-related quality of life brought about by the treatment strategies. Third, we will verify whether correlations exist between the observed changes and the baseline metabolomic profile or the concentration of inflammatory mediators. Fourth, we will evaluate whether changes differ in functional status and frailty between the older and the senior RhythmC groups. Fifth, we will assess whether there is a difference in response to the RhythmC strategy with respect to depressive symptoms, health-related quality of life, and cognition between older and senior RhythmC participants. Sixth, we will identify whether a sleep- or activity-related variable, a metabolic marker, or an inflammatory mediator concentration is associated with a lack of clinical response to sinus rhythm restoration. Finally, we will evaluate concordance among the different measures of frailty in patients with AF.

### Sample Size

In a previous pilot analysis, a small sample of patients (n=46, age 77 years, SD 7) were evaluated at baseline and at follow-up after ECV. Persistence of sinus rhythm was associated with a significant increase in the SPPB total score (from 9.6, SD 2.3, to 10.9, SD 1.6; *P*=.002), which was absent in those in whom arrhythmia had relapsed (from 8.9, SD 2.5, to 9, SD 2.6; *P*=.75) [38]. On this basis, given SDs, respectively, of 2 and 2.5, 80 RhythmC and 40 RateC participants would be necessary to detect a significant increase in physical performance under the RhythmC strategy when compared to the RateC strategy (α=.05; power=81%). Accordingly, we decided to enroll 40 participants in both the senior and the older RhythmC group (n=80 in total), as well as 40 participants in the RateC group. The estimated sample size should allow us to identify different responses among the treatment strategies and compensate for a dropout rate of 10%.

### Statistical Analysis

Continuous variables will be expressed as mean (SD) or, in the case of a nonnormal distribution, as median values and IQRs. Discrete variables will be presented as raw numbers and percentages. Continuous variables will be compared using ANOVA and the corresponding nonparametric test (ie, Kruskal-Wallis test), while the *χ*^2^ test will be used for categorical variables. Differences in patients’ clinical, instrumental, and laboratory characteristics between treatment strategies will be tested at baseline. All descriptive analyses will also check for center-related differences. In the case of a significant association of a variable with the primary outcome, all related procedures will be accordingly adjusted. Most patients undergoing the RhythmC strategy will be treated with ECV, a procedure characterized by an immediate success rate of about 90% [39] and a 2-month incidence of relapse of about 30% [40]. Participants with treatment failure will be excluded from the outcome analysis. Changes in variables between baseline and follow-up will be evaluated using general linear models (repeated-measures analysis design) or multivariable linear regression analysis models to adjust for any potential confounder, such as age, gender, body size, comorbidities, drug therapy, mood, and any other variable possibly related to imbalances. Multivariable linear regression models will also be used to explore the correlation between biochemical and inflammation-related variables and the 1-month changes in each component of the CGA. Cluster analysis will be used to identify patient variables characteristic of a homogeneous response to each AF treatment strategy. Given the relatively small number of cases, we plan not to use any technique for imputation of missing values or employ any propensity score–matching analyses. The 1-month incidence of acute urinary infections and the number of general practitioner visits not related to cardiac or respiratory reasons will be considered for sensitivity analysis. A 2-tailed *P* value <.05 will indicate statistical significance. Statistical analysis will be performed using IBM SPSS Statistics, macOS edition (version 29, 64-bit).

### Ethical Considerations

The CAFFE study was evaluated and funded as a Project of Relevant National Interest by the Italian Ministry of University and Research (PRIN, protocol ID: 2022L9NPKH). The study conforms to the Declaration of Helsinki. The protocol was initially approved by the Research Ethics Committee of the Florence Region, site of the coordinating center, on April 24, 2024. The same code assigned by the Italian Ministry was used (protocol ID 2022L9NPKH). The final approval to enroll patients was given by the General Direction of the University Hospital of Careggi, Florence, on June 4, 2024 (protocol 0013844). The Research Ethics Committees of the two other sites, Bologna and Pisa, were then informed and started their procedures, giving their final approval, respectively, on June 19, 2025 (code 400/2025/Oss/AOUBo), and on February 6, 2025 (code 26150). All patients will give their written informed consent to participate in the study after being informed of the details of the procedures, the potential benefits, and the immediate and long-term hypothetical unfavorable outcomes. However, the study is observational, and the protocol closely reflects what is usually undertaken in clinical practice; consequently, the procedural risk should be low. Because older patients will be enrolled, great care will be taken to carefully explain all phases of the protocol, even if, as previously detailed, participants with severe cognitive decline or who are unable to be assessed with the CGA will be excluded. Collection and analysis of data will be performed while ensuring patients’ anonymity.

## Results

In October 2025, 103 participants (90 with AF and 13 without AF) were enrolled in the CAFFE study; of these, 31 patients with arrhythmia were evaluated at the University of Florence coordinating center. We preliminarily present the characteristics of the latter group of participants, who had complete information in the database of the core laboratory, as follows: mean age 78, SD 9 years; 8 women (25.8%); mean BMI 26.5, SD 3.8 kg/m^2^; and mean CHA_2_DS_2_-VA score 4.2, SD 1.2. In particular, 23 (73%) participants belonged to the older RhythmC group, 6 (20%) to the senior RhythmC group, and 2 (7%) to the RateC group. Overall, cognitive, affective, and functional performance was not severely affected, with mean MMSE, GDS, and SPPB scores of 28.2, SD 2, 3.3, SD 2.5, and 8.8, SD 2.6, respectively. However, according to the FiND questionnaire, of these 13 participants, only 13 (43.3%) were robust, while 7 (20%) were frail and 11 (36.7%) were disabled.

The preliminary results we obtained show that robustness is less common in an older population with arrhythmia. Older individuals with AF are complex, as shown by a higher CHA_2_DS_2_-VA score, and for the most appropriate management of their disease, it is important to use both clinical and CGA tools. The information obtained from these tools is complementary and allows clinicians to appropriately define a patient’s phenotype [41]. From this perspective, the CAFFE study findings should contribute to identifying the most appropriate strategy to treat AF in older individuals. Our main results should be published in summer 2027.

## Discussion

### Principal Findings

The CAFFE study aims to compare the different effects of a RhythmC strategy with those of a more conservative RateC strategy in patients with AF who are 65 years or older. Importantly, the protocol will allow us to simultaneously analyze the follow-up changes in a younger (65‐74 years) and an older (≥75 years) cohort of participants undergoing RhythmC treatment and individuals without overt, uncontrolled, acute, or chronic conditions. The EAST-AFNET 4 trial recently extended the findings of the AFFIRM study, showing, in a population with a mean age of 70 years, that an early RhythmC strategy was more beneficial than a RateC strategy, reducing the incidence of cardiovascular outcomes [18,42], particularly frequent among older individuals. Interestingly, the observed benefit increased if the comorbidity burden was higher [43].

Indeed, AF is highly prevalent in the older segments of the population [1]. Notably, the progressive increase in arrhythmia prevalence in aged individuals is associated not only with all-cause mortality but also with a higher number of years spent with disability [6]. Moreover, the most elderly patients with AF are frail [44]. It is possible that this pattern is associated with the activation of chronic low-grade inflammation, with arrhythmia considered an expression of inflammaging [5,10]. The CAFFE study aims to clarify the possible benefits of AF management on the end points typical of the CGA, which is important not only in terms of health-related quality of life but also of survival.

### Study Limitations

Some limitations of the protocol should be mentioned. First is its observational design. It is known that it is particularly difficult to design and carry out a randomized controlled clinical trial in such a complex population, and it is more feasible to draw information from real-world experiences. However, we are confident that the large number of collected variables will allow us to satisfactorily describe the patients enrolled and to properly adjust the multivariable models designed to define the associations that will be found. Second, only 3 centers will participate in the study. This is due to the prespecified requirements set by the funding organization, the Italian Ministry of University and Research, which are aimed at obtaining results in a relatively short time, limiting organizational problems, and promoting effective collaboration among the units. Third, the relatively low number of participants we enrolled should be mentioned, a condition that could potentially limit the ability to adequately adjust the analysis and to assess the influence of confounders. However, as reported, the sample size evaluation that was performed based on a previous preliminary study justified our choice. Furthermore, the presence of a younger cohort of patients with AF undergoing a RhythmC strategy and a control group should also allow us to clarify, in secondary analyses, the differences we observed between treatment options in older individuals. Fourth, each center will preferentially use 1 therapeutic strategy for AF management according to its usual practice. This approach will allow us to avoid the need to randomly assign a particular form of therapy. It should be kept in mind that all 3 centers are in Italian university hospitals, that they are highly specialized, and that they follow current guideline recommendations, which do not provide strict recommendations for older individuals [11,12]. Fifth, follow-up length was set at 1 month, a time window that may potentially limit the possibility of noticing any significant therapy-related improvement. However, as previously reported, some studies showed that sinus rhythm restoration was associated with a better left ventricular performance after only a few hours [20] and that persistence of sinus rhythm completely reversed the hemodynamic consequences of arrhythmia in about 4 weeks [19]. These changes were also associated with an increase in cerebral blood flow [21]. Finally, because of the characteristics of the Italian population, a large portion of our patients will be White.

In conclusion, the CAFFE study aims to compare the benefits of a RhythmC strategy with those of a RateC strategy for AF in older individuals, those most exposed to arrhythmia and its complications. If frailty and disability are less frequent at the follow-up evaluation, this study could be the starting point for specifically designed clinical trials integrating cardiology and geriatric practice, designed to identify a strategy to improve the survival and functional profile of older individuals with AF.

## Supplementary material

10.2196/92572 Checklist 1 STROBE checklist of items that should be included in reports of observational studies.

## Abbreviations

ADL: Katz Basic Activities of Daily Living
AF: atrial fibrillation
CAFFE: Cardioversion of Atrial Fibrillation and Frailty in the Elderly
CGA: Comprehensive Geriatric Assessment
EKG: electrocardiogram
FiND: Frail Non-Disabled
GDS-15: the 15-item Geriatric Depression Scale
IADL: Lawton Instrumental Activities of Daily Living
IL-6: interleukin-6
MMSE: Mini-Mental State Examination
OPG: osteoprotegerin
SF-12: 12-item Short Form Health Survey
SPPB: Short Physical Performance Battery
TNF-α: tumor necrosis factor-α
