# Supplementary material for: Effects of Rhythm Control versus Rate Control on Atrial Fibrillation and Frailty Development in Older Patients: Protocol for the Cardioversion of Atrial Fibrillation and Frailty in the Elderly (CAFFE) Study, an Observational, Prospective Cohort Study
Source: JMIR Res Protoc. 2026 Sep 29;15:e92572. doi: 10.2196/92572 (PMC13621886; doi:10.2196/92572)
Supplement: Supplementary file 1 [file resprot-v15-e92572-s001.pdf]

STROBE Statement—checklist of items that should be included in reports of observational studies

	Item No.	Recommendation	Page No.	Relevant text from manuscript
**Title and abstract**	1	(*a*) Indicate the study's design with a commonly used term in the title or the abstract	1	Atrial Fibrillation and frailty development in older patients. Do therapeutic options exert a protective effect? The protocol of the CAFFE, an observational, prospective, cohort study
		(*b*) Provide in the abstract an informative and balanced summary of what was done and what was found	2	The CAFFE study was funded as a Research Project of National Interest by the Italian Ministry of University and Research. Ethical Committee final approval was given on June 2024. In October 2025, the coordinating center had enrolled 31/40 AF patients. All centers are now completing the enrollment. Main results should be published in summer 2027.
**Introduction**				
Background / rationale	2	Explain the scientific background and rationale for the investigation being reported	3-5	Atrial fibrillation (AF) is the most frequent sustained arrhythmia affecting older people. This condition is usually correlated with the increased risk of stroke, heart failure and dementia, and it can provide the basis for the development of frailty and disability.<br>To date, two main different approaches exist for the treatment of AF, one based on rate-control, and the other based on rhythm-control.<br>Some observations suggest that the interruption of the arrhythmia can hinder the functional decline and frailty development in an older AF population.
Objectives	3	State specific objectives, including any prespecified hypotheses	5	The project primarily aims at evaluating if rhythm-control is more effective than rate-control of the arrhythmia to hinder frailty progression and disability.

**Methods**				
Study design	4	Present key elements of study design early in the paper	5	The CAFFE ("Cardioversion of Atrial Fibrillation and Frailty in the Elderly") study is a prospective, multicenter, observational cohort study, aimed at clarifying some of the knowledge gaps regarding the management of AF in older patients, and at providing evidence to support therapeutic decisions to improve clinical outcomes and quality of life.
Setting	5	Describe the setting, locations, and relevant dates, including periods of recruitment, exposure, follow-up, and data collection	6-7, 15	The CAFFE study will enroll a cohort of 120 AF outpatients and 40 healthy control subjects during a 13-month long time window. Three centers will take part to the study. In particular, the Geriatric Cardiology Unit (University of Florence) will preferentially enroll the Older Rhythm-control Group, the Cardiology Unit (University of Bologna) the Senior Rhythm-control Group, and the Geriatric Unit (University of Pisa) the Rate-control Group patients. The Control Group population will be enrolled by all centers during their routine outpatient activity.<br>Each subject will undergo at the 1-month follow-up. Collection and analysis of data will be performed warranting patients' anonymity.
Participants	6	(*a*) *Cohort study*—Give the eligibility criteria, and the sources and methods of selection of participants. Describe methods of follow-up	6-7	To be included in the study, patients should have an EKG, a Holter EKG or an implantable device diagnosis of AF in the preceding 12 months. The exclusion criteria are a) a life expectancy <12 months, b) on-going therapy with immunosuppressant drugs, c) active cancer, d) recent (<1 month) major surgery, e) severe cognitive decline or inability to follow the therapeutic schemes, f) physical impossibility to undergo the Comprehensive Geriatric Assessment, g) permanent

				AF, h) any attempt of ablation/cardioversion of AF in the previous month, i) cachexia/extreme thinness or obesity, j) severe pre-existing frailty, and k) refusal to participate.<br>Each patient and control subject will undergo at baseline, in a fasting condition, at the standard clinical evaluation including EKG and echocardiogram. This will be followed by the Comprehensive Geriatric Assessment administration and by blood samples drawing.<br>At the 1-month follow-up, each subject will undergo to the standard clinical visit and to a repeated Comprehensive Geriatric Assessment evaluation.
		(*b*) *Cohort study*—For matched studies, give matching criteria and number of exposed and unexposed	6	The CAFFE study will enroll a cohort of N=120 AF outpatients (N=40 Older Rhythm-control Group, age ≥75 years; N=40 Senior Rhythm-control Group, age 65-74 years; N=40 Rate-control Group, age ≥65 years) and healthy control subjects (N=40 No-AF Group age ≥65 years), without structural cardiovascular diseases, arrhythmias or other relevant chronic clinical conditions.
Variables	7	Clearly define all outcomes, exposures, predictors, potential confounders, and effect modifiers. Give diagnostic criteria, if applicable	12-13	The primary endpoint of the study is: to evaluate, at the 1-month follow-up visit, if the improvement of functional status (SPPB) with the AF rhythm-control strategy is higher than that observed with rate-control.<br>The secondary endpoints of the study are:<br>i) to evaluate if the reduction of frailty with AF rhythm-control is greater than that observed with rate-control. It will be used the FiND questionnaire, the Frailty Phenotype, and the Clinical Frailty Scale.<br>ii) to evaluate the changes between baseline and follow-up of depressive symptoms (GDS), neuro-cognitive

				profile (MMSE) and health-related quality of life (SF-12) by treatment strategies;<br>iii) to verify if a correlation exists between the observed changes and the baseline metabolomic profile or the concentration of inflammatory mediators (IL-6, OPG, TNF-α);<br>iv) to evaluate the existence of different changes of functional status and frailty between the Older and the Senior rhythm-control groups;<br>v) to assess a different response to the rhythm-control strategy related to depressive symptoms, health-related quality of life and cognition between Older and Senior rhythm-control subjects;<br>vi) to identify a sleep or activity-related variable, a metabolic "marker", or an inflammatory mediator concentration associated with lack of a clinical response to sinus rhythm restoration;<br>vii) to evaluate the concordance among the different measures of frailty in AF patients.<br>Potential confounders: center-related differences.
Data sources / measurement	8*	For each variable of interest, give sources of data and details of methods of assessment (measurement). Describe comparability of assessment methods if there is more than one group		Clinical and instrumental variables related to cardiologic and internal medicine evaluation are widely standardized among centers.<br>Several meetings between Geriatric and Cardiology centers will be held to make homogeneous the administration of the Comprehensive Geriatric Assessment tools: the ADL, the IADL, the SPPB, the FiND questionnaire, the Frailty Phenotype, the Clinical Frailty Scale, the GDS, the MMSE and the SF-12.
Bias	9	Describe any efforts to address potential sources of bias	14	Trend differences of variables between baseline and the end of the follow-up will be evaluated with General Linear Models (repeated measures analysis design) or

				with multivariable linear regression analysis models in order to adjust for any potential confounder, such as age, gender, body size, comorbidities, drug therapy, mood and any other variable possibly related to unbalances.
Study size	10	Explain how the study size was arrived at	13-14	In a pilot analysis, a small series of patients was evaluated after ECV. Persistence of sinus rhythm was associated with a significant increase of SPPB total score, absent in those in whom the arrhythmia relapsed. On this basis, given a standard deviation, of 2.0 and 2.5, N=80 rhythm-control and N=40 rate-control subjects would be necessary to detect a significant increase of physical performance with the rhythm-control strategy when compared to a rate-control one (alpha=0.05; power=81%).

Quantitative variables	11	Explain how quantitative variables were handled in the analyses. If applicable, describe which groupings were chosen and why	14	Continuous variables will be expressed as mean $\pm$ SD or, in the case of a not normal distribution, as median values and interquartile ranges. Discrete variables will be presented as raw numbers and percentages.
Statistical methods	12	(*a*) Describe all statistical methods, including those used to control for confounding	14-15	1) Continuous variables will be compared using analysis of variance and the related non-parametric tests, while chi-squared test will be used for categorical variables.<br>2) Trend differences of variables between baseline and the end of the follow-up will be evaluated with General Linear Models (repeated measures analysis design) or with multivariable linear regression analysis models in order to adjust for any potential confounder (i.e., age, gender, body size, comorbidities, drug therapy, mood, etc.).

				3) Multivariable linear regression analysis models will be also used to explore the correlation between biochemical and inflammation-related variables and the 1-month changes of each component of the CGA.<br>4) Cluster analysis will be used to identify patients' variables characteristic of a homogeneous response to each AF treatment strategy.
		(*b*) Describe any methods used to examine subgroups and interactions	14	Center-related differences in patients' characteristics between treatment strategies will be tested at baseline. In the case of a significant association for a variable correlated to the primary outcome, all the related procedures will be accordingly center adjusted.
		(*c*) Explain how missing data were addressed	14	Given the relatively small number of cases, we planned not to use any technique for imputation of missing values
		(*d*) *Cohort study*—If applicable, explain how loss to follow-up was addressed	14	The estimated population size should allow to compensate for a drop-out rate of 10%.
		(*e*) Describe any sensitivity analyses	14-15	The one-month incidence of acute urinary infections and the number of the general practitioner medical visits not related to heart or respiratory reasons were considered for sensitivity analysis.
**Results**				
Participants	13*	(a) Report numbers of individuals at each stage of study—eg numbers potentially eligible, examined for eligibility, confirmed eligible, included in the study, completing follow-up, and analysed		Not applicable
		(b) Give reasons for non-participation at each stage		Not applicable
		(c) Consider use of a flow diagram		Not applicable

Descriptive data	14*	(a) Give characteristics of study participants (eg demographic, clinical, social) and information on exposures and potential confounders		Not applicable
		(b) Indicate number of participants with missing data for each variable of interest		Not applicable
		(c) *Cohort study*—Summarise follow-up time (eg, average and total amount)		Not applicable
Outcome data	15*	*Cohort study*—Report numbers of outcome events or summary measures over time		Not applicable
Main results	16	(*a*) Give unadjusted estimates and, if applicable, confounder-adjusted estimates and their precision (eg, 95% confidence interval). Make clear which confounders were adjusted for and why they were included		Not applicable
		(*b*) Report category boundaries when continuous variables were categorized		Not applicable
		(*c*) If relevant, consider translating estimates of relative risk into absolute risk for a meaningful time period		Not applicable
Other analyses	17	Report other analyses done—eg analyses of subgroups and interactions, and sensitivity analyses		Not applicable
**Discussion**				
Key results	18	Summarize key results with reference to study objectives	16	The CAFFE Study aims to compare the different effects of a rhythm-control strategy with those of a more conservative rate-control one in AF patients aged ≥65 years. Importantly, the protocol will allow to simultaneously analyze the follow-up changes of a younger (65-74 years) and an older (≥75 years) cohort of subjects undergoing a rhythm control approach, and of individuals without overt, uncontrolled, acute or chronic conditions.

Limitations	19	Discuss limitations of the study, taking into account sources of potential bias or imprecision. Discuss both direction and magnitude of any potential bias	17-18	Some limitations of the protocol are to be mentioned. 1) Its observational design. It is known that it is particularly difficult to project and to carry out a randomized controlled clinical trial in a so complex population, and it is more convenient to draw information from "real world" experiences.<br>2) Only three centers will participate to the study. This is due to the pre-specified requirements that were given by the funding organization, the Italian Ministry of University and Research, aimed at obtaining results in a relatively short time, limiting problems related to organization and promoting an effective collaboration among the units.<br>3) The relatively low number of subjects we enrolled is to be mentioned, a condition potentially restricting the possibility to adequately adjust the analysis and to assess the influence of confounders.<br>4) Each center will preferentially follow one therapeutic strategy for AF management, according to its usual practice. This behavior will allow us to overcome the need to randomly assign a particular form of therapy.<br>5) Follow-up length was set at 1 month, a time-window potentially limiting the possibility to notice any significant therapy-related improvement.<br>6) Because of the characteristics of the Italian population, great part of our patients will be Caucasian.
Interpretation	20	Give a cautious overall interpretation of results considering objectives, limitations, multiplicity of analyses, results from similar studies, and other relevant evidence	18	The CAFFE study is aimed to compare the benefits of an AF rhythm-control strategy with those of a rate-control one in older individuals, the most exposed to the arrhythmia and to its complications.

Generalizability	21	Discuss the generalizability (external validity) of the study results	18	If frailty and disability were less frequent at the follow-up evaluation, this experience could be the starting point for specifically addressed clinical trials, designed to identify a strategy to improve the survival and the functional profile of older AF individuals.
**Other information**				
Funding	22	Give the source of funding and the role of the funders for the present study and, if applicable, for the original study on which the present article is based	18-19	The CAFFE Study has been funded with 200.250 Euro as a Project of Relevant National Interest by the Italian Ministry of University and Research (PRIN, Protocol id: 2022L9NPKH). The Ministry does not and will not exert any influence on the study conduct.
